# Gut Hormones and Postprandial Metabolic Effects of Isomaltulose vs. Saccharose Consumption in People with Metabolic Syndrome

**DOI:** 10.3390/nu17152539

**Published:** 2025-08-01

**Authors:** Jiudan Zhang, Dominik Sonnenburg, Stefan Kabisch, Stephan Theis, Margrit Kemper, Olga Pivovarova-Ramich, Domenico Tricò, Sascha Rohn, Andreas F. H. Pfeiffer

**Affiliations:** 1 Department of Endocrinology, The Second Affiliated Hospital, School of Medicine, Zhejiang University, Hangzhou 310009, China; 2 Department of Endocrinology, Diabetes and Nutrition, Charité—Universitätsmedizin Berlin, Corporate Member of Freie Universität Berlin, Humboldt-Universität zu Berlin, 12203 Berlin, Germany; stefan.kabisch@charite.de; 3 Department of Clinical Nutrition, German Institute of Human Nutrition Potsdam-Rehbrücke, Arthur-Scheunert-Allee 114-116, 14558 Nuthetal, Germany; sonnenburg@uni-potsdam.de (D.S.); dr.m.k@gmx.de (M.K.); olga.ramich@dife.de (O.P.-R.); 4 German Center for Diabetes Research (Deutsches Zentrum für Diabetesforschung e.V.), Ingolstädter Landstraße 1, 85764 Neuherberg, Germany; 5 BENEO-Institute, c/o BENEO GmbH, Wormser Str. 11, 67283 Obrigheim, Germany; stephan.theis@beneo.com; 6 Department of Molecular Metabolism and Precision Nutrition, German Institute of Human Nutrition Potsdam-Rehbruecke, Arthur-Scheunert-Allee 114-116, 14558 Nuthetal, Germany; 7 Department of Clinical and Experimental Medicine, University of Pisa, 56126 Pisa, Italy; domenico.trico@unipi.it; 8 Institute of Food Technology and Food Chemistry, Technische Universität Berlin, Gustav-Meyer-Allee 25, 13355 Berlin, Germany; rohn@tu-berlin.de

**Keywords:** isomaltulose, saccharose, incretin hormones, metabolic syndrome, second meal effect, preload timing

## Abstract

**Background:** Low-glycemic index (GI) carbohydrates like isomaltulose (ISO) are known to enhance incretin release and to improve postprandial glucose control at the following meal (an effect known as second meal effect, or SME), which is particularly beneficial for individuals with metabolic syndrome (MetS). This study aimed to assess the most effective preprandial interval of ISO- or saccharose (SUC) snacks (1 h vs. 3 h preload) to enhance prandial incretin responses to a subsequent meal. **Methods:** In a randomized crossover design, 15 participants with MetS completed four experimental conditions on four non-consecutive days, combining two preload types (ISO or SUC) and two preload timings (Intervention A: 3 h preload; Intervention B: 1 h preload). Specifically, the four conditions were (1) ISO + Intervention A, (2) SUC + Intervention A, (3) ISO + Intervention B, and (4) SUC + Intervention B. The order of conditions was randomized and separated by a 3–7-day washout period to minimize carryover effects. On each study day, participants consumed two mixed meal tests (MMT-1 and MMT-2) with a standardized preload (50 g ISO or SUC) administered either 3 h or 1 h prior to MMT-2. Blood samples were collected over 9 h at 15 predefined time points for analysis of glucose, insulin, *C*-peptide, and incretin hormones (GLP-1, GIP, and PYY). **Results:** The unique digestion profile of ISO resulted in a blunted glucose ascent rate (ΔG/Δt: 0.28 vs. 0.53 mmol/L/min for SUC, *p* < 0.01), paralleled by synonyms PYY elevation over 540 min monitoring, compared with SUC. ISO also led to higher and more sustained GLP-1 and PYY levels, while SUC induced a stronger GIP response. Notably, the timing of ISO consumption significantly influenced PYY secretion, with the 3 h preload showing enhanced PYY responses and a more favorable SME compared to the 1 h preload. **Conclusions:** ISO, particularly when consumed 3 h before a meal (vs. 1 h), offers significant advantages over SUC by elevating PYY levels, blunting the glucose ascent rate, and sustaining GLP-1 release. This synergy enhances the second meal effect, suggesting ISO’s potential for managing postprandial glycemic excursions in MetS.

## 1. Introduction

Metabolic syndrome (MetS) is a cluster of interrelated conditions—specifically insulin resistance/impaired glucose tolerance, hypertension, elevated triglyceride levels, low high-density lipoprotein (HDL) cholesterol levels, and central obesity, which are linked to an increased risk of type 2 diabetes mellitus (T2DM) and cardiovascular disease [1]. Effective management of postprandial glucose levels is essential for individuals with MetS. Dietary interventions that lower the glycemic index (GI) of consumed foods have shown promise in improving glucose control [2]. Carbohydrates with a medium GI like saccharose (SUC; GI = 55–70) induce rapid spikes in blood glucose and insulin, whereas low-GI carbohydrates such as isomaltulose (ISO; GI = 32) are digested more slowly, producing stable postprandial glucose profiles [3,4,5]. This metabolic advantage positions ISO as a promising alternative to SUC [6]. Its unique α-1,6-glycosidic bond resists hydrolysis by intestinal sucrase-isomaltase, delaying glucose absorption and underpinning both its low glycemic index and sustained energy release properties. These biochemical characteristics not only explain ISO’s steady glucose delivery but also highlight its clinical relevance for glycemic management in populations with metabolic disorders.

Previous studies demonstrated that low glycemic meals not only improve postprandial metabolic response directly after consumption but may also limit the postprandial glycemic and insulinemic response to the subsequent meal, which is known as the “second meal effect” (SME). The SME has been shown to be beneficial for glycemic control and is associated with a reduction in metabolic and cardiovascular risks [7,8]. It may also explain the detrimental acute metabolic effect of long meal distances as practiced by meal skipping and intermittent fasting, despite their positive impact on overall energy intake [9,10,11].

The incretin hormones glucose-dependent insulinotropic polypeptide (GIP) and glucagon-like peptide-1 (GLP-1) regulate insulin and glucagon after their nutrient-induced secretion from the gut. Moreover, GLP-1 receptor agonists, as well as GLP-1/GIP dual agonists, have become a more important strategy in the treatment of T2DM and obesity [12,13,14]. The peptide tyrosine tyrosine (PYY, 1–36), a gut hormone that has attracted less attention, is co-secreted with GLP-1 by L-cells in the distal small intestine and also in the colon [15], which is converted by DPP-4 to PYY (3–36) upon release. PYY has been increasingly recognized for its role in enhancing satiety and modulating glucose metabolism [16]. The endogenous release of GLP-1 and PYY is mediated by a variety of stimuli including bile acids, fat, protein, carbohydrates, and short-chain fatty acids resulting from the degradation of fibers including resistant starch and low-digestible carbohydrates [17,18]. In addition, in mice, the secretion of PYY entero-hormone after a meal has been proposed as a trigger for ileal secretion of GLP-1 [19].

The mechanism of action behind SME has been so far poorly understood, particularly regarding the role of gut hormones such as GLP-1 and PYY in modulating postprandial metabolic responses. While emerging evidence suggests that sustained incretin activity may mediate SME, critical gaps remain in explaining how these hormones interact with dietary variables like carbohydrate timing. For instance, the influence of preload intervals (e.g., 1 h vs. 3 h) on incretin-driven SME remains largely unexplored, despite its potential to optimize glycemic control through strategic nutrient scheduling. This lack of mechanistic clarity highlights the need for targeted studies to unravel how temporal patterns of carbohydrate intake synergize with incretin dynamics to amplify or attenuate SME. While the role of GLP-1 in SME has been extensively studied, the contribution of PYY—a potent anorexigenic hormone—remains under-investigated. To our knowledge, this is the first study designed to directly compare distinct preload timing (1 h vs. 3 h) for ISO/SUC, specifically targeting its unique capacity to stimulate PYY secretion—a less-explored incretin—and thereby amplify SME in MetS. This temporal dimension represents a critical innovation in optimizing functional carbohydrate timing for people with MetS.

Consequently, this study aimed to compare the metabolic effects of ISO vs. SUC and to assess the most effective pre-prandial intervals of ISO- or SUC-containing snacks (1 h vs. 3 h preload) to enhance prandial incretin responses to a subsequent meal. We propose that ISO’s metabolic benefits extend beyond its low glycemic index, with its prolonged preload timing uniquely enhancing PYY secretion and SME. Given the novel focus on preload timing and PYY-mediated mechanisms, this proof-of-concept study was designed with a preliminary sample size to generate hypotheses for future validation in larger cohorts.

## 2. Materials and Methods

### 2.1. Ethics and Study Design

This clinical trial is a prospective, randomized, double-blind, controlled nutritional intervention study in adults with metabolic syndrome. It was carried out in the Department of Clinical Nutrition at the German Institute of Nutrition (DIfE), Potsdam-Rehbruecke, Germany, and was reviewed and approved by the Ethics Committee Charité—Universitätsmedizin Berlin, Berlin, Germany. The study was registered in ClinicalTrials.gov (No. NCT03806920). The recruitment spanned 9 months between October 2016 and June 2017 to identify eligible participants, and the study was primarily completed in September 2018.

**Figure A1:**
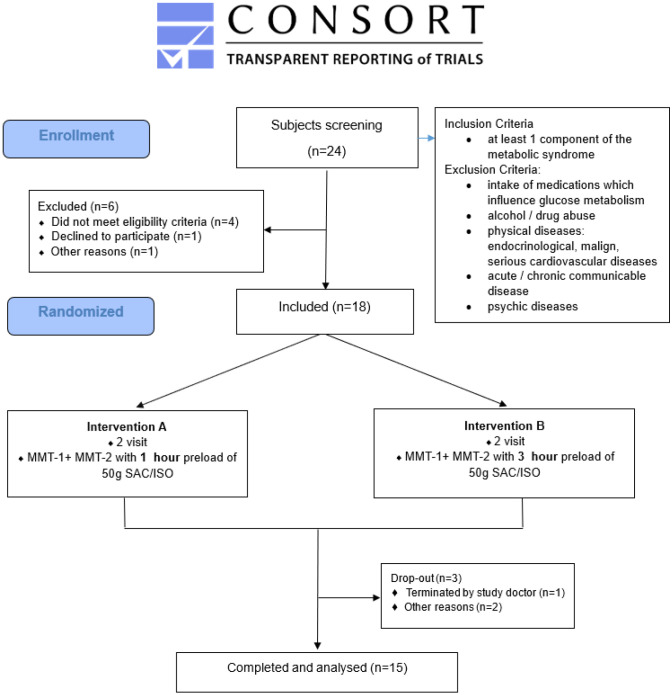
Consort form.

Twenty-four patients were screened, and fifteen participants were included; the detailed inclusion and exclusion criteria are presented in the Consort Diagram (Figure A1). Oral glucose tolerance test (OGTT) administering 75 g glucose was performed to confirm normal glucose tolerance for all subjects, and participants who were eligible for this study performed interventions A and B with either 50 g ISO or SUC on 4 non-consecutive days. Their characteristics and clinical parameters are presented in Table 1. All participants provided written informed consent prior to the study.

**Figure 1:**
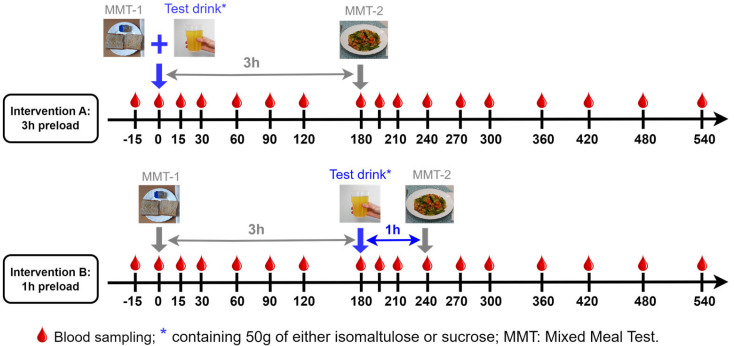
The design overview of Intervention A (3 h preload) and B (1 h preload). Blood samples were collected at 15 time points (arrows) over 540 min. Data analysis included both individual time-point measurements and AUC calculations (0–540 min) for glucose, insulin, *C*-peptide, GLP-1, GIP, and PYY.

The overall study design is presented in Figure 1. For Intervention A (3 h preload), all subjects received in the morning the first mixed meal tests (MMT-1) plus 50 g of either ISO (isomaltulose (Palatinose™), BENEO GmbH, Mannheim, Germany) or SUC (saccharose/sucrose, Südzucker AG, Mannheim, Germany) in the form of a citrus drink (500 mL), followed by the second mixed meal test (MMT-2) after 3 h. For Intervention B (1 h preload), subjects consumed MMT-1 followed by MMT-2 with a 1 h preload of either 50 g SUC or ISO (the same sugars as in Intervention A). The compositions of both MMTs are presented in Table A1.

On each clinical investigation day, an antecubital catheter was inserted into a forearm vein of a participant for blood collection. Blood samples were drawn twice at fasting state (−15 and 0 min) and then at the time intervals 15, 30, 60, 90, 120, 180, 195, 210, 240, 270, 300, 360, 420, 480, and 540 min with either 1 h or 3 h ISO/SUC preload before MMT-2 (Figure 1). The blood was collected into pre-chilled EDTA-coated tubes containing DPP-4 inhibitor (2.5 mM, Merck KGaA, Darmstadt, Germany) for determining GIP, GLP-1, and PYY and into inhibitor-free EDTA-tubes for analyzing insulin and *C*-peptide as well as routine clinical parameters. Immediately after the blood collection, serum samples were allowed to clot for 10 min at room temperature while plasma samples containing EDTA and/or DPP-4 were centrifuged immediately for 10 min at 4 °C. Supernatant was stored as aliquots after centrifugation at −80 °C for further analysis. The measurement of all biomarkers was performed after the study was completed in September 2018.

### 2.2. Biomarkers Measurements

Blood glucose concentrations were measured with a glucometer (Optium Xceed; Abbott Laboratories Inc., Abbott Park, IL, USA); clinical routine parameters were measured with ABX Pentra 400 (HORIBA Europe GmbH, Oberursel, Germany). Total as well as percentage fat mass and fat-free mass were determined using both BOD POD (Body Composition System; Life Measurement Inc., Concord, CA, USA). Plasma GLP-1 and GIP were determined by the electrochemiluminescent method with MSD (Meso Scale Diagnostics LLC, Gaithersburg, MD, USA); plasma PYY was measured using an ELISA assay (Millipore, Burlington, MA, USA); insulin and *C*-peptide were measured by an ELISA immunoassay (Mercodia, Uppsala, Sweden). The sensitivity and specificity of those assays has been published previously [20]. The analytical validity of incretin measurements was confirmed using established protocols [21].

### 2.3. Calculations and Statistical Analysis

Mean fasting concentrations (glucose, insulin, and *C*-peptide) were calculated as the mean of the two fasting samples. HOMA-IR was calculated based on insulin as described before [22]. Areas under the curve (AUC, 0–540 min) were calculated by the trapezoidal rule. The Insulin Sensitivity Index according to Matsuda (ISI Matsuda) was used for the evaluation of whole-body insulin sensitivity during OGTT [23].

The distribution of variables was evaluated by Shapiro–Wilk test. For the analysis of the difference between time courses (0–540 min), the repeated measures ANOVA (rmANOVA) was performed using the Greenhouse-Geisser correction if sphericity was not given. Comparisons between the interventions were made using the paired *t*-test or Wilcoxon test, depending on distribution. To compare the groups, either the Mann–Whitney U-Test or Student’s unpaired *t*-test was used, depending on the distribution of data. Results are described as means ± SD in tables and means ± SEM in graphs, and statistical significance is defined as *p* < 0.05. All statistical calculations were performed using SPSS 28.0 (SPSS Inc., Chicago, IL, USA). The graphs were generated by GraphPad Prism 8 (San Diego, CA, USA).

## 3. Results

Fifteen subjects (9 males and 6 females, age 61.9 ± 8.5 years) completed the study. Baseline characteristics of each group are shown in Table 1.

### 3.1. Metabolic Parameter Responses to ISO/SUC Preload

#### 3.1.1. Glucose

**Figure 2:**
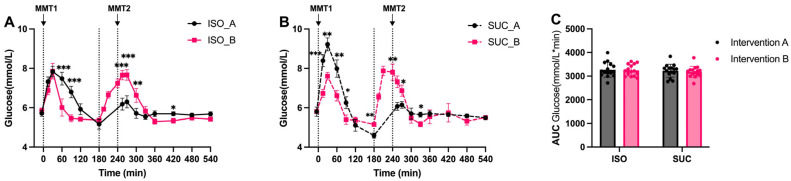
Glucose responses to MMTs with ISO (solid line, (**A**)) and SUC (dotted line, (**B**)) preload in Intervention A and B, AUC (0–540 min) glucose responses in Intervention A and B (**C**). Data are described as mean ± SEM, * *p* < 0.05, ** *p* < 0.01, *** *p* < 0.001. ISO_A/SUC_A: 3 h preload; ISO_B/SUC_B: 1 h preload.

**Figure 3:**
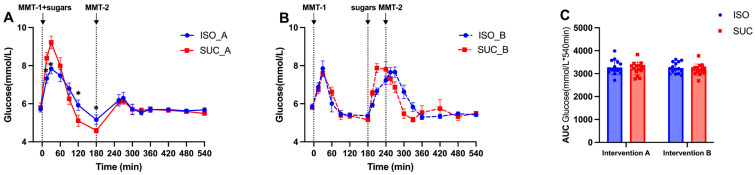
Glucose responses to MMTs with ISO (blue) and SUC (red) preload in Intervention A (**A**) and B (**B**), AUC (0–540 min) glucose responses to ISO (blue) and SUC (red) in Intervention A and B (**C**). Data are described as Mean ± SEM. * *p* < 0.05.

In both interventions, ISO demonstrated a lower and delayed increase in blood glucose levels compared to SUC. Specifically, during MMT-1 in Intervention A, ISO consumption resulted in a slower rise in glucose levels, with a delayed peak and a smoother decline. In contrast, SUC led to a more rapid glucose spike followed by a significant drop (Figure 2A and Figure 3A). This pattern was observed under both preload conditions, but being more pronounced in Intervention B. While the overall AUC glucose did not differ significantly between ISO and SUC, the time-course analysis revealed significant differences in the rate of glucose increase and the peak glucose concentration (Figure 2C and Figure 3C). The 22% reduction in peak glucose is a key finding and is consistent with the blunted glucose ascent rate.

Overall, ISO was more effective in maintaining stable glucose levels with a longer preload interval, indicating that a 3 h preload improves postprandial glucose control compared to the 1 h preload. There were no significant differences in glucose concentrations observed between SUC and ISO across the combined measurement periods, as confirmed by AUC glucose (0–540 min) (Figure 2C and Figure 3C).

#### 3.1.2. Insulin

**Figure 4:**
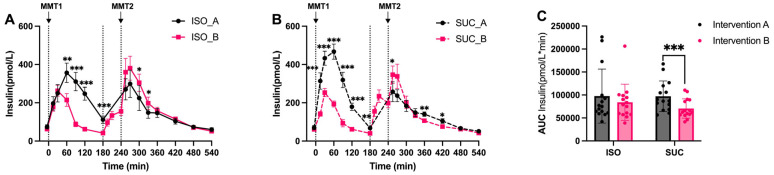
Insulin responses to MMTs with ISO (solid line, (**A**)) and SUC (dotted line, (**B**)) preload in Intervention A and B, AUC (0–540 min) Insulin responses in Intervention A and B (**C**). Data are described as mean ± SEM, * *p* < 0.05, ** *p* < 0.01, *** *p* < 0.001.

**Figure 5:**
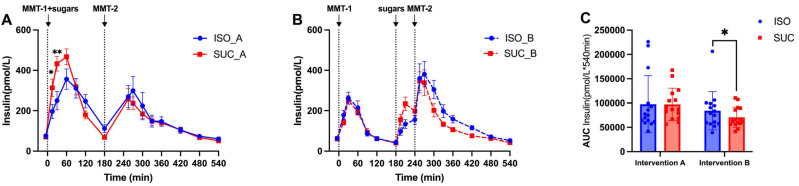
Insulin responses to MTMTs with ISO (blue) and SUC (red) preload in Intervention A (**A**) and B (**B**), AUC (0–540 min) glucose responses to ISO (blue) and SUC (red) in Intervention A and B (**C**). Data are described as Mean ± SEM. * *p* < 0.05, ** *p* < 0.01.

**Figure 6:**
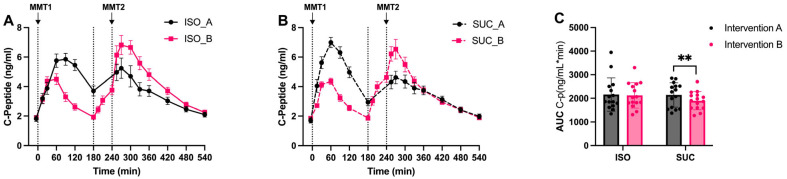
*C*-peptide responses to MMTs with ISO (solid line, (**A**)) and SUC (dotted line, (**B**)) preload in Intervention A and B, AUC (0–540 min) *C*-peptide responses in Intervention A and B (**C**). Data are described as mean ± SEM, ** *p* < 0.01.

**Figure 7:**
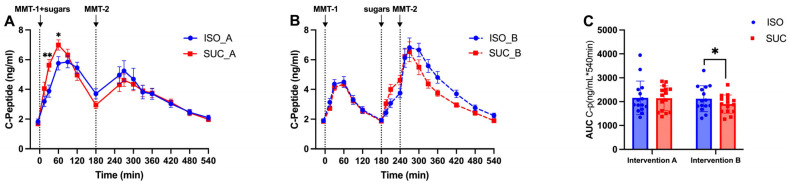
*C*-peptide responses to MMTs with ISO (blue) and SUC (red) preload in Intervention A (**A**) and B (**B**), AUC (0–540 min) glucose responses to ISO (blue) and SUC (red) in Intervention A and B (**C**). Data are described as Mean ± SEM, * *p* < 0.05, ** *p* < 0.01.

The insulin (Figure 4A–C and Figure 5A–C) and *C*-peptide (Figure 6A–C and Figure 7A–C) responses mirrored the glucose patterns, with ISO leading to a more sustained insulin release compared to SUC across both interventions (Figure 5A). In Intervention A, ISO resulted in a lower initial insulin peak but maintained higher insulin levels over time, suggesting improved insulin sensitivity, while SUC triggered a rapid insulin spike at 60 min, followed by a return to basal levels by 180 min (Figure 4A and Figure 5A,B).

These results indicate that ISO seems to demand more stable insulin secretion over a longer period, especially in Intervention A, while SUC tended to cause rapid insulin fluctuations, particularly in the shorter preload interval (Figure 4C and Figure 5C).

#### 3.1.3. Gut Hormones (GIP, GLP-1, PYY)

**Figure 8:**
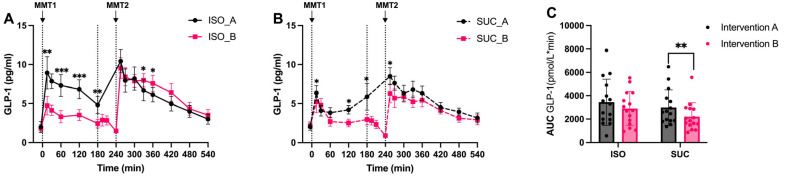
GLP-1 responses to MMTs with ISO (solid line, (**A**)) and SUC (dotted line, (**B**)) preload in Intervention A and B, AUC (0–540 min) GLP-1 responses in Intervention A and B (**C**). Data are described as mean ± SEM, * *p* < 0.05, ** *p* < 0.01, *** *p* < 0.001.

**Figure 9:**
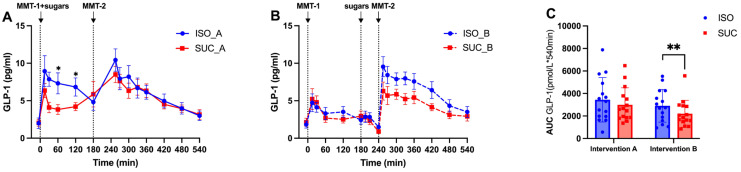
GLP-1 responses to MMTs with ISO (blue) and SUC (red) preload in Intervention A (**A**) and B (**B**), AUC (0–540 min) glucose responses to ISO (blue) and SUC (red) in Intervention A and B (**C**). Data are described as Mean ± SEM, * *p* < 0.05, ** *p* < 0.01.

In Intervention A (3 h preload), both SUC and ISO increased GLP-1 concentrations significantly in the first 15 min of MMT-1 consumption (*p* < 0.01 vs. baseline) (Figure 8A,B and Figure 9A,B). However, ISO maintained higher GLP-1 levels over time compared to SUC during the 60–180 min period, contributing to improved glucose metabolism and insulin release (Figure 9A). During MMT-2, no significant differences in GLP-1 concentrations were observed between the two preloads (Figure 8B and Figure 9B).

In Intervention B (1 h preload), delayed application of the DPP-4 inhibitor at 195, 210, and 240 min precluded reliable GLP-1/GIP quantification at these time points due to potential analyte degradation. Consequently, statistical comparisons for these hormones were restricted to Intervention A and earlier time points in Intervention B (Figure 8C). PYY data remained unaffected as it is DPP-4-insensitive. Trend-level consistency in GLP-1 responses between interventions (Figure 9A,B) supports our conclusions regarding ISO’s effects on distal gut hormones in Intervention B. GLP-1 measurements were not fully reliable due to delayed DPP-4 inhibitor application, but trends suggested a similar GLP-1 response with ISO showing a more sustained elevation.

When comparing the effects of different preload timing of ISO/SUC, SUC induced a significantly stronger GLP-1 response (Figure 8C) with higher AUC values compared to Intervention B (*p* < 0.05). This suggests that a 3 h preload optimizes the capacity of SUC to stimulate early-phase GLP-1 secretion, likely due to sufficient time for nutrient sensing and L-cell activation in the distal gut.

**Figure 10:**
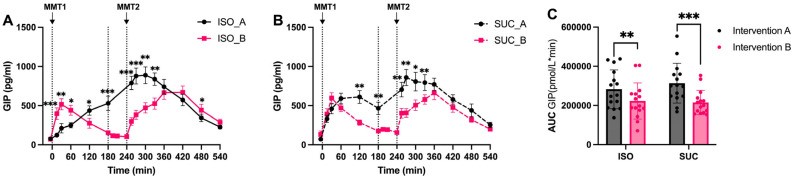
GIP responses to MMTs with ISO (solid line, (**A**)) and SUC (dotted line, (**B**)) preload in Intervention A and B, AUC (0–540 min) GIP responses in Intervention A and B (**C**). Data are described as mean ± SEM, * *p* < 0.05, ** *p* < 0.01, *** *p* < 0.001.

**Figure 11:**
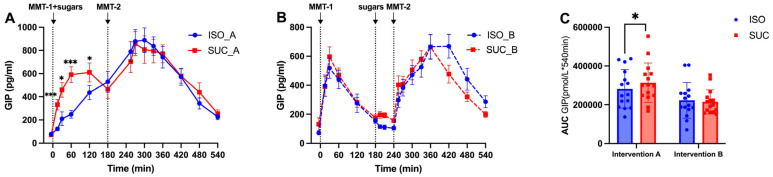
GIP responses to MMTs with ISO (blue) and SUC (red) preload in Intervention A (**A**) and B (**B**), AUC (0–540 min) glucose responses to ISO (blue) and SUC (red) in Intervention A and B (**C**). Data are described as Mean ± SEM, * *p* < 0.05, *** *p* < 0.001.

GIP responses differed notably between the interventions. SUC resulted in stronger GIP levels in both interventions, particularly in the early phases of MMT-1 (Figure 10A,B and Figure 11A,B). ISO, on the other hand, led to lower GIP levels, consistent with its slower digestion and absorption profile (Figure 11A). In Intervention A, SUC induced a rapid GIP peak at 30 min (*p* < 0.001, Figure 11A). This pattern persisted in Intervention B with SUC showing higher early-phase GIP secretion (0–60 min, *p* < 0.01). The total response calculated with AUC confirmed significantly greater integrated GIP responses for SUC in both Intervention A (*p* < 0.001) and Intervention B (*p* < 0.01) (Figure 10C). And SUC induced higher GIP secretion in Intervention A than in Intervention B (Figure 11C).

**Figure 12:**
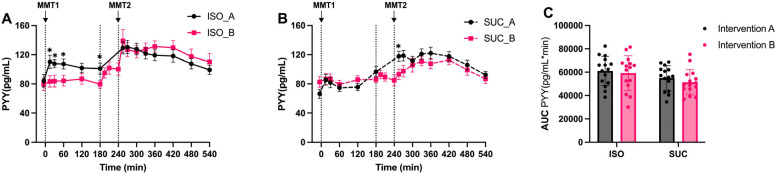
PYY responses to MMTs with ISO (solid line, (**A**)) and SUC (dotted line, (**B**)) preload in Intervention A and B, AUC (0–540 min) PYY responses in Intervention A and B (**C**). Data are described as mean ± SEM, * *p* < 0.05.

**Figure 13:**
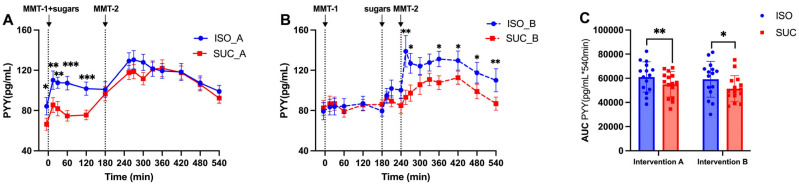
PYY responses to MMTs with ISO (blue) and SUC (red) preload in Intervention A (**A**) and B (**B**), AUC (0–540 min) glucose responses to ISO (blue) and SUC (red) in Intervention A and B (**C**). Data are described as Mean ± SEM, * *p* < 0.05, ** *p* < 0.01, *** *p* < 0.001.

PYY responses followed a similar pattern to GLP-1, with ISO consistently maintaining higher PYY levels compared to SUC, in both Intervention A and Intervention B (Figure 12A and Figure 13A). Overall, ISO showed a more favorable incretin profile, with sustained GLP-1 and PYY responses both (*p* < 0.001) and Intervention B (*p* < 0.01) (Figure 13C).

## 4. Discussion

ISO was reported to induce an attenuated and delayed increase in glucose and insulin immediately after consumption, leading to a favorable metabolic regulation postprandial compared to SUC in people with MetS in several studies [24]. Various studies stated that high and fluctuating glucose levels are a major risk factor in the development of insulin resistance and altered insulin secretion, which may result in T2DM via progressive β-cell dysfunction. One review also showed that the consumption of a high GI food leads to postprandial hyperglycemia and increased NEFA concentrations, causing β-cell disorder via glucotoxicity or a lipotoxic reaction and oxidative stress in obesity and T2DM diabetes [7].

This study was a randomized crossover design controlling for inter-individual variability, comprehensive 9 h metabolic profiling at 15 time points, and novel investigation of temporal effects of preload timing on PYY-mediated second meal phenomena. Meanwhile, the differential effects of ISO and SUC on incretin hormone secretion are particularly noteworthy. The present study identified that ISO significantly increased GLP-1 levels, whereas SUC led to a more pronounced GIP response, particularly in Intervention A. This finding is consistent with the existing literature on the role of GLP-1 in enhancing insulin sensitivity and promoting glucose homeostasis [18]. GLP-1 not only stimulates insulin secretion but also inhibits glucagon release, slows gastric emptying, and promotes satiety, making it a key hormone in the regulation of postprandial glucose levels [25,26,27]. The sustained GLP-1 response observed with ISO may also contribute to SME, wherein the metabolic response to a subsequent meal is improved due to the prolonged presence of incretins. This has important implications for dietary strategies aiming at improved glycemic control throughout the day. The SME observed in the present study suggests that ISO consumption, particularly with a 3 h preload, may help stabilize blood glucose levels over extended periods, thereby reducing the risk of glycemic excursions that contribute to insulin resistance and T2DM progression.

This is the first human evidence demonstrating that preload timing critically shapes ISO-driven PYY/GLP-1 co-secretion and SME efficacy. The prolonged PYY elevation observed with ISO may originate from its slow digestion kinetics. Our study provides novel mechanistic insights by demonstrating that a 3 h preload interval is critical for maximizing ISO-induced PYY release and SME efficacy—a previously unreported temporal dependency. This aligns with ISO’s slow digestion kinetics, enabling distal L-cell activation where it enhances PYY/GLP-1 co-secretion via SGLT1-mediated glucose sensing and SCFA receptor activation (e.g., FFAR2/3)—a mechanism supported by murine models [18], though direct human evidence is needed [19]. The present findings support incorporating ISO into snack formulations as an alternative sweetening agent consumed 3 h before main meals. For instance, replacing SUC in afternoon tea biscuits with ISO could enhance glycemic control at dinner—a high-risk period for postprandial hyperglycemia in metabolically compromised individuals. The potential clinical applications of these findings are significant. So ISO could be an alternative where SUC is technically needed for food technology or food structure (e.g., cakes, marmalade, food conservation, etc.). In the afternoon, snacks consumed 3 h before dinner with ISO, as a low-GI carbohydrate, may reduce postprandial hyperglycemia at the subsequent meal—a high-risk period for glucose excursions in MetS or those at risk of developing T2DM. The ability of ISO to enhance GLP-1 secretion and sustain its effects could make it a valuable dietary tool for reducing postprandial glycemic variability—a critical factor in the management of diabetes and its complications. Moreover, the observed differences in the metabolic effects of ISO and SUC underscore the importance of carbohydrate quality in dietary planning. While both carbohydrates provide similar caloric content, their impact on glycemic and insulinemic responses is markedly different. This highlights the need for clinicians and dietitians to consider not just the quantity but also the quality of carbohydrates in the diets of individuals with metabolic disorders.

However, there are certain limitations of the present study. It had a relatively small group of subjects, with a total of fifteen subjects. A limitation was the technical error in DPP-4 inhibitor application during Intervention B, which compromised GLP-1/GIP measurements at select time points. In addition, the technical error was obtained as part of intervention B. Due to a time-delayed application of the DPP-4 at 195, 210, and 240 min, the measurements for GLP-1 and GIP showed disproportionately low concentrations in all four interventions. Nevertheless, the consideration of the concentration ratios allowed clear conclusions to be drawn from the concentration curves. The consistent trends across valid time points and robust PYY data support our conclusions regarding ISO’s effects on distal gut hormones. Additionally, our comparison of preload timings involves confounding factors: the differing meal intervals (1 h vs. 3 h preload) and administration contexts (preload with MMT-1 vs. between meals). While reflecting real-world conditions, these differences limit isolation of timing effects; future studies should employ fixed meal intervals with mid-interval preloads (e.g., 1 h vs. 3 h post-MMT-1) to better isolate temporal mechanisms. Despite these constraints, the robust PYY elevation with ISO—particularly at 3 h—supports our key finding of timing-dependent SME optimization.

Furthermore, the mechanisms underlying the differential effects of ISO and SUC on incretin hormone secretion remain to be fully elucidated. While the present study provides evidence of a stronger GLP-1 response with ISO, the precise pathways through which ISO modulates incretin release and its downstream effects on glucose metabolism need further exploration. Investigating the role of other gut hormones, such as PYY and the interplay between GLP-1 and GIP, could provide deeper insights into the metabolic benefits of low-GI carbohydrates.

## 5. Conclusions

In conclusion, a 3 h preload of ISO synergistically enhances PYY and GLP-1 release and SME via distal gut signaling, offering a practical dietary strategy to prime postprandial glycemic excursions from sugar-rich foods. This supports the benefit of ISO as a functional SUC substitute in timed snack interventions for MetS management. Critically, the 3 h preload ISO optimizes postprandial metabolism by sustaining PYY levels and amplifying SME—a novel finding with significant implications for dietary approaches in metabolic syndrome. Future research should investigate the long-term impact of ISO consumption on sustained glycemic control, insulin sensitivity, and overall metabolic health, including effects on inflammation, lipid profiles, and cardiovascular risk.

## Figures and Tables

**Table 1:** Clinical characteristics for all participants at screening.

Characteristics (*n* = 15)	Mets
Male:Female	9:6
Age (years)	61.9 ± 8.5
BMI (kg/m^2^)	30.8 ± 4.0
WHR	0.96 ± 0.1
Fat mass (%)	31.8 ± 17.0
Fat-free mass (%)	61.1 ± 7.2
HbA1c (%)	5.6 ± 0.1
HOMA-IR	2.9 ± 0.2
Fasting glucose (mmol/L)	5.8 ± 0.5
Fasting insulin (pmol/L)	60.9 ± 23.0
Fasting *C*-peptide (pmol/L)	2.0 ± 0.4

HbA1c: glycated hemoglobin A1c; BMI: body mass index; WHR: waist-to-hip ratio; HOMA-IR: Homeostatic Model Assessment for Insulin Resistance; Data are described as mean values ± SD.

## Data Availability

To protect the privacy of the participants, the data collected for this study cannot be shared publicly. However, the data are available upon reasonable request from the first and the corresponding authors.

## Abbreviations

The following abbreviations are used in this manuscript:

AUC	Area Under the Curve
DPP-4i	Dipeptidyl Peptidase-4 inhibitor
FFAR2/3	Free Fatty Acid Receptor 2/3
GI	Glycemic Index
GIP	Glucose-dependent Insulinotropic Polypeptide
GLP-1	Glucagon-Like Peptide-1
HOMA-IR	Homeostatic Model Assessment for Insulin Resistance
ISO	Isomaltulose
MetS	Metabolic Syndrome
MMT	Mixed Meal Test
OGTT	Oral Glucose Tolerance Test
PYY	Peptide YY
SUC	Saccharose
SCFA	Short-Chain Fatty Acids
SGLT1	Sodium-Glucose Cotransporter-1
SME	Second Meal Effect
T2DM	Type 2 Diabetes

## Appendix A

**Table A1:** The composition of Standardized Test Meals.

	Quantity (g)	Energy (kcal)	CH (g)	F (g)	P (g)	Fiber (g)
**Dinner the evening before**						
Chicken meal (Frosta)	500	520	62.0	14.0	32.5	7.5
**Breakfast fixed component (MMT-1)**						
Toast whole grain (REWE)	50	128	24.0	2.0	4.0	1.5
Butter (Meggle)	17	126	0.0	14.0	0.1	0.0
**Total**	67	254	24.0	16.0	4.1	1.5
**Lunch (MMT-2)**						
“Spätzle” meal (Frosta)	500	530	65.0	14.0	33.0	9.0
Olive oil (Henry Lamotte)	15	133	0.0	15.0	0.0	0.0
**Total**	515	663	65.0	29.0	33.0	9.0
**Test Meal (ISO/SUC)**						
Citrus drink (BENEO)	400	202.5	50.0	0.0	0.0	0.0
